# Antidepressant and Antiaging Effects of Açaí (*Euterpe oleracea* Mart.) in Mice

**DOI:** 10.1155/2019/3614960

**Published:** 2019-07-24

**Authors:** José Rogério Souza-Monteiro, Gabriela P. F. Arrifano, Ana Isabelle D. G. Queiroz, Bruna S. F. Mello, Charllyany S. Custódio, Danielle S. Macêdo, Moisés Hamoy, Ricardo S. O. Paraense, Leonardo O. Bittencourt, Rafael R. Lima, Rommel R. Burbano, Hervé Rogez, Cristiane F. Maia, Barbarella M. Macchi, José Luiz M. do Nascimento, Maria Elena Crespo-López

**Affiliations:** ^1^Laboratory of Molecular Pharmacology, Institute of Biological Sciences, Federal University of Pará, Belém, Brazil; ^2^Laboratory of Experimental Neuropathology, Department of Pharmacology, University of Oxford, Oxford OX1 3QT, UK; ^3^Laboratory of Neuropharmacology, Department of Physiology and Pharmacology, Federal University of Ceará, Fortaleza, Brazil; ^4^Laboratory of Pharmacology and Toxicology of Natural Products, Institute of Biological Sciences, Federal University of Pará, Belém, Brazil; ^5^Laboratory of Structural and Functional Biology, Institute of Biological Sciences, Federal University of Pará, Belém, Brazil; ^6^Laboratory of Molecular Biology, Ophir Loyola Hospital, Belém, Pará, Brazil; ^7^Centre for Valorisation of Amazonian Bioactive Compounds (CVACBA) and Federal University of Pará, Belém, Brazil; ^8^Laboratory of Pharmacology of Inflammation and Behavior, Institute of Health Sciences, Federal University of Pará, Belém, PA, Brazil; ^9^Laboratory of Molecular and Cellular Neurochemistry, Institute of Biological Sciences, Federal University of Pará, Belém, Brazil

## Abstract

Depression is a mental disorder that affects 300 million people of all ages worldwide, but fewer than half of those with the condition receive adequate treatment. In addition, the high pharmacological refractoriness (affecting 30%-50% of patients) and toxicity of some classical antidepressants support the pursuit of new therapies. People with this condition show depressed mood, loss of pleasure, high levels of oxidative stress, and accelerated biological aging (decreased telomere length and expression of the telomerase reverse transcriptase (TERT), the enzyme responsible for telomere maintenance). Because of the close relationship between depression and oxidative stress, nutraceuticals with antioxidant properties are excellent candidates for therapy. This study represents the first investigation of the possible antidepressant and antiaging effects of commercial samples of clarified açaí (*Euterpe oleracea*) juice (EO). This fruit is rich in antioxidants and widely consumed. In this study, mice were treated with saline or EO (10 *μ*L/g, oral) for 4 days and then with saline or lipopolysaccharide (0.5 mg/kg, i.p.) to induce depressive-like behavior. Only four doses of EO were enough to abolish the despair-like and anhedonia behaviors and alterations observed in electromyographic measurements. The antidepression effect of EO was similar to that of imipramine and associated with antioxidant and antiaging effects (preventing lipid peroxidation and increasing TERT mRNA expression, respectively) in three major brain regions involved in depression (hippocampus, striatum, and prefrontal cortex). Additionally, EO significantly protected hippocampal cells, preventing neuronal loss associated with the depressive-like state and nitrite level increases (an indirect marker of nitric oxide production). Moreover, EO alone significantly increased TERT mRNA expression, revealing for the first time a potent antiaging action in the brain that suggests neuroprotection against long-term age-related consequences.

## 1. Introduction

Depression is a mental disorder that represents an important and growing problem in public health, with an estimated 300 million people of all ages affected worldwide [1]. Although the pharmacological arsenal for depression includes selective serotonin reuptake inhibitors (SSRIs), such as fluoxetine, or tricyclic antidepressants, such as imipramine, the World Health Organization estimates that less than half of the patients receive adequate treatment [1]. In some countries, that rate is less than 10% [1]. In addition to difficulties with adequate access to health care, a main problem in the treatment of depression is pharmacological refractoriness, which affects 30%-50% of people with the condition [2]. Side effects of some classical antidepressants make it urgent to seek new therapies that could serve as adjuvants or less-toxic alternatives.

Depression is characterized by symptoms such as depressed mood, loss of interest or pleasure, feelings of guilt or low self-esteem, and sleep or appetite disorders, creating a significant impact on quality of life [1]. Accelerated aging also has been demonstrated in patients with depression characterized by a significant decrease in telomere length and expression of telomerase reverse transcriptase (TERT), the enzyme responsible for telomere maintenance [3–6]. This effect may especially affect the brain increasing its susceptibility to age-related disorders.

Many areas of the brain are involved in mood regulation [7–10] and thus relevant to the neurobiology of major depressive disorder (MDD) and depressive-like symptoms [11]. Among the main symptoms present in people with MDD, learning and memory problems are related to hippocampal damage, and reward and emotional behaviors are associated with alterations in the prefrontal cortex and striatum [11].

Clinical and preclinical studies have confirmed the close relationship between MDD and the imbalance of increased oxidative stress and decreased antioxidant defenses [12–15]. Oxidative and nitrosative stress play a crucial role in the pathophysiology of unipolar and bipolar depression [16]. In parallel to oxidative stress, immunoinflammatory mechanisms of depression have been described [17]. Based on the inflammatory/oxidative stress component of depression, a mouse model of depressive-like behavior induced by lipopolysaccharide (LPS) has been developed. After 24 hours of a single low exposure to LPS, the animals show depressive-like alterations such as anhedonia and despair-like behavior [18]. This model has translational validity based on recent findings that depressed patients have higher plasma LPS levels [19].

Although analysis of isolated compounds from natural sources for the development of new antidepressant drugs is important, the study of nutraceuticals (a food, part of a food, vitamin, mineral, or herb that provides health benefits) is also essential to uncover potential nutritional strategies. A key reason is that these products can be realistic alternatives and adjunctive therapies for depression in isolated populations or people living in developing and low-income countries.

In this context, Amazonia biodiversity provides a special opportunity. Açaí is a fruit (of a very common palm in the Amazon basin, *Euterpe oleracea* Martius, family Arecaceae) commonly consumed in the Amazon and largely available on the international market [20, 21]. This fruit, or the drink made from it, is a well-recognized functional food. Called a “superfruit” because of its antioxidant properties, açaí has attracted increasing attention from the global nutraceutical market [21, 22]. Its biological activities, such as antiparasitic, anticarcinogenic, or metabolic action have been described [23–25]. Moreover, recent preclinical data support a potent neuroprotective effect of açaí [26]. In the latter study, consumption of commercial samples of clarified açaí juice significantly reduced tonic-clonic seizures and their consequences [26], probably because of the action on the gabaergic system [27].

Despite this pronounced neuroprotective effect, *in vivo* studies with açaí are scarce, especially those related to neuropsychiatric disorders. In the last decade, however, some phytochemical compounds such as ellagic acid and apigenin have shown efficacy in preventing depressive-like behaviors [28–30], and many of them are found in açaí.

The aim of this study was to investigate for the first time the possible antidepressant-like and antiaging effects of commercial samples of clarified açaí juice (EO).

## 2. Materials and Methods

### 2.1. Animals and Ethical Aspects

Male Swiss mice (20-30 g) were maintained in standard environmental conditions (22 ± 1°C, humidity 60 ± 5%, and 12 h light-dark cycle) with food and water *ad libitum*. All experimental procedures were approved by the Committee for Ethics in Experimental Research with Animals of the Federal University of Pará (license number 89-15) and followed the guidelines of the NIH Guide for the Care and Use of Laboratory Animals.

### 2.2. Clarified Açaí (*Euterpe oleracea* Mart.) Juice (EO)

Samples of EO were kindly provided by Amazon Dreams (Belém, Pará, Brazil) and produced by a patented process (PI 1003060-3). Briefly, clarified açaí was prepared from fresh fruits of *E. oleracea* Martius (Arecaceae). After cleaning the fruits, pulping was performed with the addition of 0.5 L of water per kilogram of fruits. The juice was subsequently microfiltered (Souza-Monteiro et al., 2005; [27]). EO is a thin, translucent, wine-colored liquid with no lipids, proteins, or fibers but rich in phenolic compounds (>1400 mg gallic acid equivalents/L). The major phenolic compounds of EO were previously analyzed using two UHPLC-DAD methods and found to be cyanidin 3-rutinoside (450 mg/L), orientin (380 mg/L), taxifolin deoxyhexose (310 mg/L), homoorientin (250 mg/L), and cyanidin 3-glucoside (180 mg/L) [27].

### 2.3. Treatments

**Figure 1:**
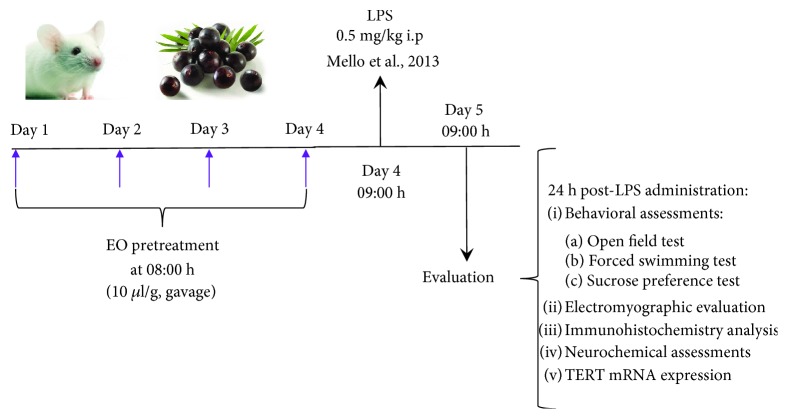
Experimental design of the study. EO, clarified juice of *Euterpe oleracea*; i.p., intraperitoneal; LPS, lipopolysaccharide.

The animals were orally treated with clarified açaí or saline (10 *μ*L/g body weight) daily for 4 days (Figure 1). Thirty minutes after the last dose, a set of animals received also imipramine (5 or 10 mg/kg, i.p.). At 24 hours following this dose, animals were treated with a single dose of saline or LPS at 0.5 mg/kg, i.p. to induce by 24 hours the depressive-like behavior that has been described previously [18]. All reagents were obtained from Sigma-Aldrich Corp., St. Louis, USA.

One set of animals was used for the analysis of spontaneous locomotor activity, despair-like behavior, neurochemical evaluations, and mRNA expression. After behavior assessments, all animals were sacrificed by cervical dislocation, and the prefrontal cortices, hippocampi, and striata were stored at -20°C (part of the tissue was previously frozen in liquid nitrogen and the other part maintained in RNAlater solution; Thermo Fisher Scientific, São Paulo, Brazil), until analysis.

Electromyographical analysis, immunohistochemical evaluations, and the study of anhedonia-like behavior were performed separately in different sets of animals as described below.

### 2.4. Behavioral Evaluations

Twenty-four hours after the administration of LPS, behavioral evaluations (mobility in open-field and forced swimming or sucrose preference test) were performed.

#### 2.4.1. Assessment of Spontaneous Locomotor Activity (Open-Field Test)

The animals were placed into an open-field arena (30 × 30 × 15 cm) with white walls and a black floor, divided into nine squares of equal areas. Spontaneous locomotor activity was observed for 5 minutes, and the number of crossed quadrants (crossings), total distance, rearings, and groomings were recorded [31].

#### 2.4.2. Assessment of Despair-Like Behavior (Forced Swim Test)

After the open-field test, the animals were placed in an acrylic transparent cylinder (25 cm height and 10 cm diameter) containing water at 22-24°C. After 1 minute of habituation, immobility time (in seconds) was recorded for a period of 5 minutes. Immobility was defined as an absence of active behaviors such as swimming, jumping, rearing, or diving [32, 33]. Animals presenting difficulties keeping their heads above water were removed and excluded.

#### 2.4.3. Assessment of Anhedonia Behavior (Sucrose Preference Test)

The presence of anhedonia behavior was tested in a different set of animals as described by Mao et al. [34]. At 48 hours before LPS administration (concomitant with pretreatment with EO or saline), animals were habituated to the consumption of 1% (*w*/*v*) sucrose solution for 24 hours. Then, the sucrose solution was replaced by water for an additional 24 hours. Animals were deprived of water and food before LPS administration. Twenty-four hours later, they were individually housed in cages with free access to two bottles, one containing water and the other containing 1% sucrose solution. Volumes consumed of each bottle were recorded after 1 hour, and the sucrose preference was calculated as follows:

(1) $$\begin{array}{c} \text{Sucrose preference}=\frac{\text{sucrose consumption}}{\text{water consuption}+\text{sucrose consuption}}\times 100\%. \end{array}$$

### 2.5. Electromyographic Evaluations

For additional evaluation of the despair/scape-like behavior, we performed electromyographic measurements. After the treatments, stainless-steel-conjugated electrodes were implanted in the semitendinosus muscle of the limbs of a subgroup of animals to acquire electromyographic records, with a recording time of 5 minutes for each animal. Electrodes were connected to a high-impedance amplifier (Grass Technologies, P511), monitored by an oscilloscope (Protek, 6510). The entire analysis was performed inside a Faraday cage. Data were continuously scanned at a rate of 1 kHz by a computer equipped with a data acquisition board (National Instruments, Austin, TX) and processed through specialized software (LabVIEW express).

The amplitude graphs show the potential difference between the reference and the electrode. A total of 1000 samples per second were observed. The spectrograms were calculated using a Hamming window with 256 points (256/1000 s), and each frame was generated with an overlap of 128 points per window. For each frame, the spectral power density (SPD) was calculated by the Welch average periodogram method. The frequency histogram was generated by the first PSD calculation of the signal using the Hamming window with 256 points, without overlap, with the PSD, resulting in a histogram constructed with boxes of 1 Hz.

### 2.6. Neurochemical Assessments

#### 2.6.1. Evaluation of Lipid Peroxidation

Lipid peroxidation was assayed by evaluating thiobarbituric acid-reactive substances [35]. Briefly, samples were slowly thawed and homogenized in Tris-HCl solution. Aliquots of these homogenates were mixed with 10% trichloroacetic acid and 0.67% thiobarbituric acid. They were then heated in a boiling water bath for 15 minutes and immediately placed in ice. Absorbance at 532 nm was recorded and compared to that of standard solutions of malonaldehyde (MDA). Lipid peroxidation was expressed as micromoles of MDA/g tissue.

#### 2.6.2. Assay of Nitrite Levels

The production of nitric oxide was indirectly evaluated by the nitrite levels in the tissue as described by Green [36]. Aliquots of the homogenates were incubated at room temperature for 10 minutes with equal volumes of the Griess solution (1% sulfanilamide in 1% H_3_PO_4_ : 0.1% N-(1-naphthyl)-thylenediamine dihydrochloride : distilled water; 1 : 1 : 1) at room temperature for 10 minutes. The absorbance was recorded at 560 nm and compared to those of standard solutions of sodium nitrite. Nitrite levels were expressed as micromoles of nitrites per milligram of tissue.

### 2.7. TERT mRNA Expression

TERT mRNA expression was determined by two-step quantitative reverse transcription PCR (RT-qPCR) using the TaqMan Gene Expression Assay, as described by Silva et al. [37]. Briefly, total RNA was extracted with the Tri Reagent (Applied Biosystems, USA) following the manufacturer's instructions. A NanoDrop spectrophotometer (Kisker, Germany) was used to evaluate the RNA concentration and quality along with 1% agarose gels. The synthesis of complementary DNA was performed using the High-Capacity cDNA Reverse Transcription Kit (Applied Biosystems, Poland).

RT-qPCR was performed using StepOnePlus equipment (Applied Biosystems, Brazil) with TaqMan® Universal PCR Master Mix and TaqMan probes (Applied Biosystems, Brazil). The GAPDH gene was selected as an internal control for RNA input and reverse transcription efficiency. All qRT-PCR reactions were run in triplicate with a final volume of 10 *μ*L for the target gene (TERT: Hs00972656_m1) and the internal control (GAPDH: NM_002046.3), for 40 cycles, using the standard cycling conditions of the machine.

Relative quantification of the gene expression was calculated by the *ΔΔ*Ct method and expressed as the fold change proposed by Livak and Schmittgen [38]. In the present study, the control group was designated as the calibrator.

### 2.8. Immunohistochemistry Analysis for Hippocampal Mature Neurons

Animals were deeply anesthetized by intraperitoneal injection of ketamine and xylazine solution for subsequent transcardiac perfusion. First, saline solution (pH 7.4) heparinized 0.9% and then paraformaldehyde (Sigma-Aldrich, USA) 4% were used in the perfusion system for fixation of the brains. Then, the brains were removed and postfixed for 4 hours in Bouin's solution with dehydration and clarification in hydroalcoholic solutions with increasing concentrations and xylol. The specimens were subsequently embedded in Paraplast® (McCormick Scientific, USA). The coronal sections of the anterior hippocampus were sliced on a microtome at 5 *μ*m of thickness. For immunohistochemical assays, the slides were pretreated with poly-D-lysine (Sigma-Aldrich) prior to the placement of sections.

Sections were immunolabeled for mature neurons (anti-NeuN: 1 : 100, Millipore). For this purpose, tissue antigen sites were recovered by heating at 60°C in citrate buffer for 20 minutes and cooled naturally. For the inhibition of the endogenous peroxidase, slides were treated with hydrogen peroxide solution in methanol for 20 minutes. After three consecutive 10-minute washes in phosphate-buffered saline- (PBS-) Tween (Sigma-Aldrich), the sections were incubated in 10% normal horse serum in PBS for 2 hours. Sections were then incubated with the primary antibody diluted in PBS previously coated overnight, with subsequent washes in PBS-Tween, and incubated with the biotinylated secondary antibody, diluted in PBS for 2 hours. They were washed again in PBS-Tween to be incubated with an avidin-biotin-peroxidase complex (ABC Kit, Vector Laboratories, USA) for 1 hour. Sections were then exposed in DAB for 40 seconds, dehydrated in alcohol and xylol, and coverslipped. Some slides were counterstained with hematoxylin for histological delimitations. More details about this protocol have been described previously [39–41].

The photomicrographs were taken with a Moticam system coupled to a Nikon Eclipse 50i optic photomicroscope. For quantification of positive cells for anti-NeuN, we used the light optical microscope Nikon Eclipse E2000 with a 1 mm^2^ grid in the eyepiece, under 40x objective magnification. In the dorsal hippocampus, three fields from each region (CA1, CA3, and dentate gyrus) were counted, as described elsewhere [39–41].

### 2.9. Statistical Analyses

Statistical analyses were performed using GraphPad Prism (version 5.0). The normality and homoscedasticity of the data were verified using the D'Agostino-Pearson test and the Bartlett test, respectively. Data are presented as mean and standard deviation (SD) and were analyzed using one-way analysis of variance followed by Tukey's post hoc test, when appropriate. *P* < 0.05 was considered significant for all analyses.

## 3. Results

**Figure 2:**
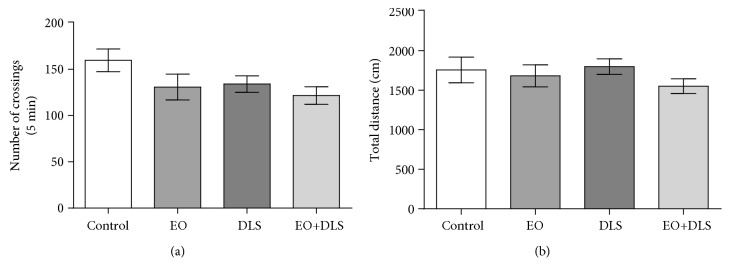
Spontaneous locomotor activity of mice in a depressive-like state (DLS) and/or treated with clarified açaí (EO): number of crossings in 5 min (a) and total distance covered (b). Data are presented as mean ± SD (*n* = 10). No significant difference was detected.

EO treatment did not interfere with the spontaneous locomotor activity, as demonstrated by the number of crossings and the total distance traveled in the open-field test (Figure 2).

**Figure 3:**
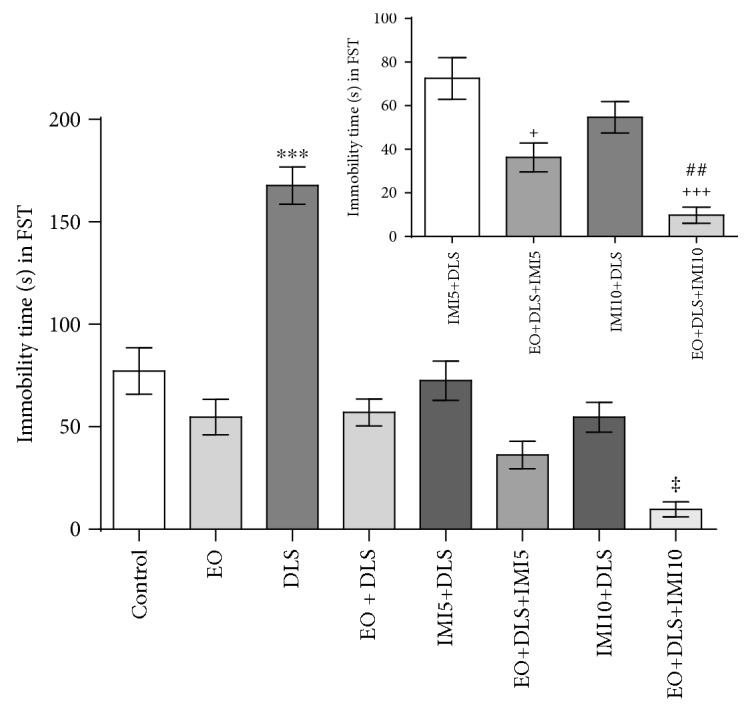
Despair-like behavior (immobility time) in the forced swim test of mice in a depressive-like state (DLS) and/or treated with clarified açaí (EO) and/or imipramine (5 or 10 mg/kg, IMI5 or IMI10, respectively). The inset shows the statistical analysis when only four groups were considered. Data are presented as mean ± SD (*n* = 5). ^∗∗∗^*P* < 0.001 vs. all groups, ^‡^*P* < 0.05 vs. control, ^+^*P* < 0.05 and ^+++^*P* < 0.001 vs. IMI5 + DLS, and ^##^*P* < 0.01 vs. IMI10+LPS.

As expected, 24 hours after LPS exposure the immobility time in the forced swim test increased significantly, indicating a depressive-like state (DLS) (Figure 3), which EO treatment reduced to control levels. Moreover, when groups treated with imipramine (given as IMI in the graphs) were compared separately (Figure 3, inset), significant differences between the therapeutic treatment with imipramine (the IMI5+DLS and IMI10+DLS groups) and the treatments with both EO and IMI (the EO+DLS+IMI5 and EO+DLS+IMI10 groups) were detected. Although additional experiments are necessary, this fact may point to a certain degree of synergism between EO and imipramine. Despite this possible synergism, the antidespair effect of EO treatment alone was as effective as that of imipramine.

**Figure 4:**
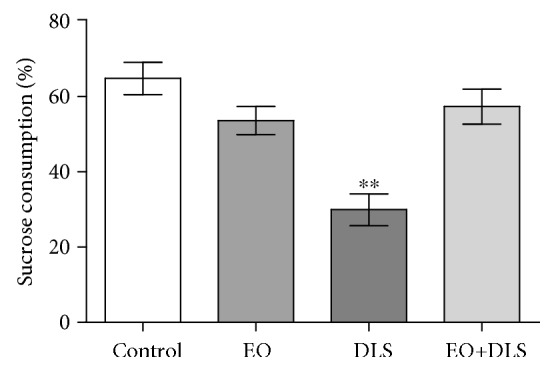
Anhedonia behavior (reduced sucrose consumption) of mice in a depressive-like state (DLS) and/or treated with clarified açaí (EO). Data are presented as mean ± SD (*n* = 13). ^∗∗^*P* < 0.01 vs. all groups.

In the sucrose preference test, animals showed a significant decrease in sucrose consumption (anhedonia behavior) caused by LPS administration, corroborating the depression-like state of the animals (Figure 4). Again, EO treatment completely prevented the anhedonia-like state induced by LPS, supporting the potent antidepressant effect of this treatment.

**Figure 5:**
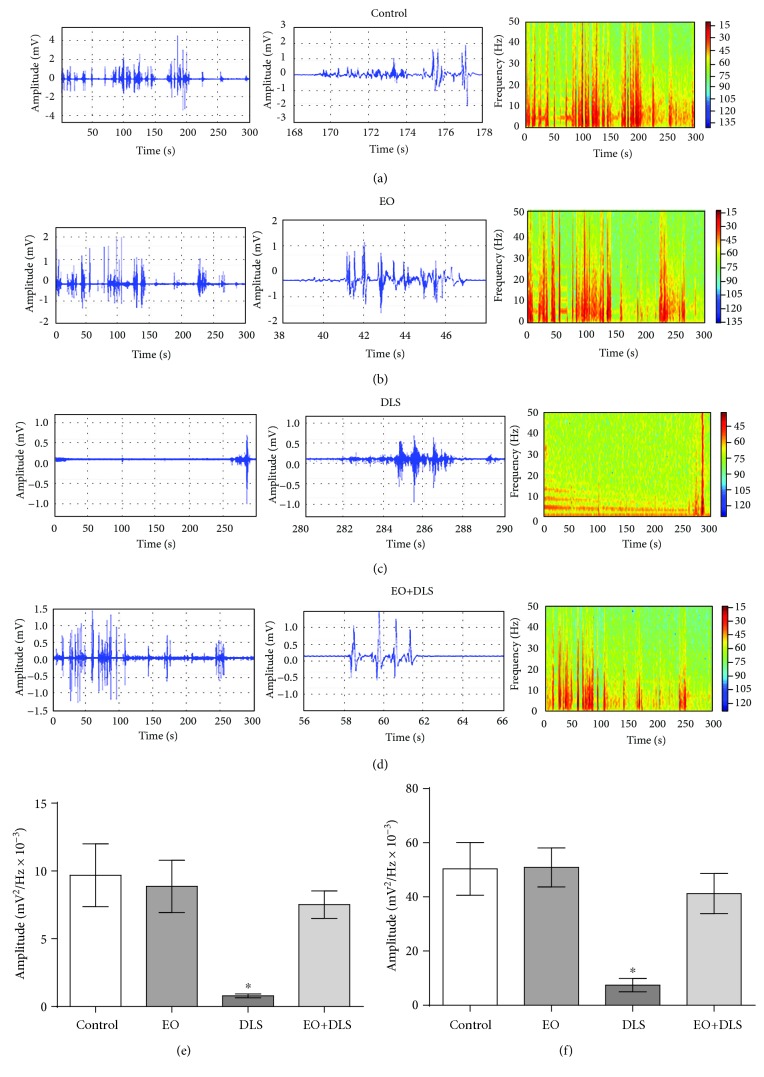
Electromyographic recordings and energy distributions (a-d), amplitude distributions (e), and amplitudes of the maximum muscular activity (f) of the frequencies up to 50 Hz recorded for 5 min in mice in a depressive-like state (DLS) and/or treated with clarified açaí (EO). Data in (e) and (f) are presented as mean ± SD (*n* = 5). ^∗^*P* < 0.05 vs. all groups.

As an additional approach to assessing the despair/scape-like behavior, we performed electromyographic measurements with 5-minute recordings of the muscular responses to stimulus from the electrode implantation. Control and EO-treated animals showed energy levels up to 50 Hz (Figure 5) corresponding to normal muscle activity. However, LPS administration caused a significant decrease in muscle activity that was entirely prevented by EO. In fact, the muscular activity of the animals that received both LPS and EO treatments was similar to that of control animals.

The results of the forced swim test and electromyography together show that EO prevented immobility or the absence of response to stimulus, representing a significant improvement in one of the more characteristic symptoms of depressive-like behavior.

**Figure 6:**
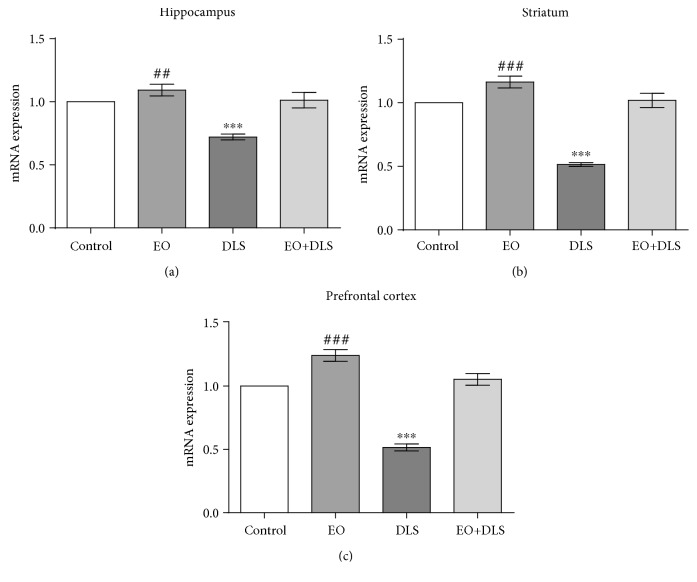
Relative expression of telomerase reverse transcriptase (TERT) mRNA in the hippocampus (a), striatum (b), and prefrontal cortex (c) of mice in a depressive-like state (DLS) and/or treated with clarified açaí (EO). Data are presented as mean ± SD (*n* = 5‐6). ^∗∗∗^*P* < 0.001 vs. all groups and ^###^*P* < 0.001 and ^##^*P* < 0.01 vs. control.

The animals with depressive-like behavior (as confirmed by the behavioral tests) presented a significant decrease (as low as 49%) of the expression of TERT in these three areas that was completely reversed by EO treatment (Figure 6). Of interest, EO alone significantly increased the expression of TERT mRNA in the three brain areas evaluated (Figure 6).

**Figure 7:**
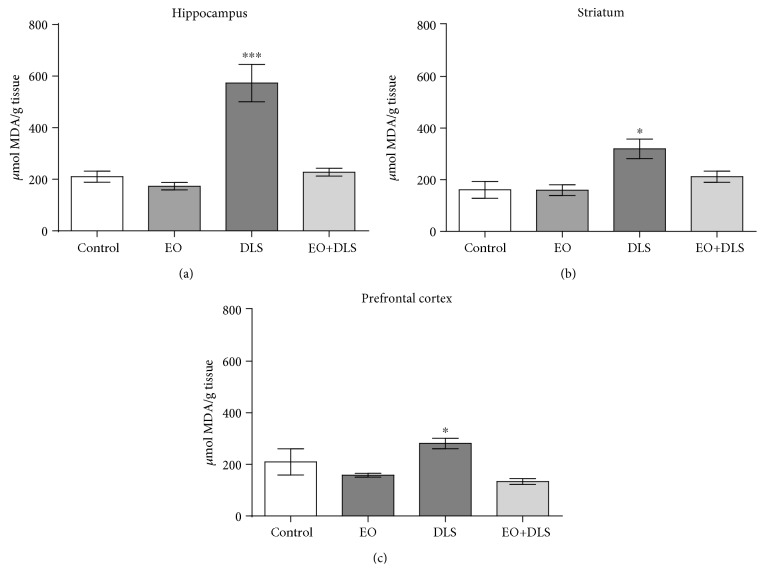
Lipid peroxidation in the hippocampus (a), striatum (b), and prefrontal cortex (c) of mice in a depressive-like state (DLS) and/or treated with clarified açaí (EO). Data are presented as mean ± SD of *μ*mol of malonaldehyde (MDA)/g tissue (*n* = 6‐10). ^∗^*P* < 0.05 and ^∗∗∗^*P* < 0.001 vs. all groups.

When thiobarbituric acid-reactive substances were analyzed to evaluate oxidative stress burden, mice with depressive-like behavior (as confirmed by the behavioral tests) presented high levels of lipid peroxidation in the three examined brain areas (Figure 7). The hippocampus was the most affected, with about two times the levels of lipid peroxidation when compared to control values. Despite this pronounced prooxidant state of the hippocampus, EO treatment totally reverted this scenario, eliminating the presence of lipid peroxidation products (Figure 7).

The hippocampus was the area most affected by the lipid peroxidation; oxidative stress is a known inducer of telomere shortening and aging, and the presence of TERT was reduced, and it is essential to protect against this deleterious effect. Given these findings, we analyzed their association with neuronal death, one of the most serious cellular consequences of oxidative stress and a probable cause, at least in part, for the depressive-like behavior.

**Figure 8:**
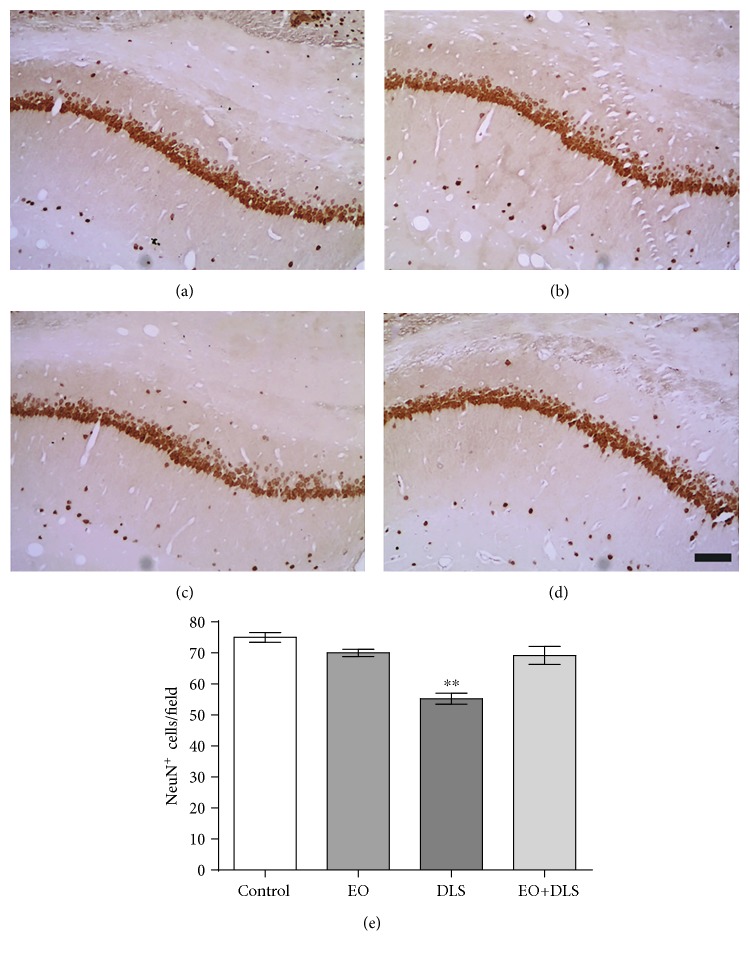
NeuN-positive cells in the CA1 region of the hippocampus of mice in a depressive-like state (DLS) and/or treated with clarified açaí (EO). Representative microphotographs of control (a), EO (b), DLS (c), and DLS+EO (d) samples and quantitation of NeuN-positive cells (e). Data are presented as mean and SD (*n* = 4‐5). ^∗∗^*P* < 0.01 vs. all groups. Scale bar: *μ*m.

**Figure 9:**
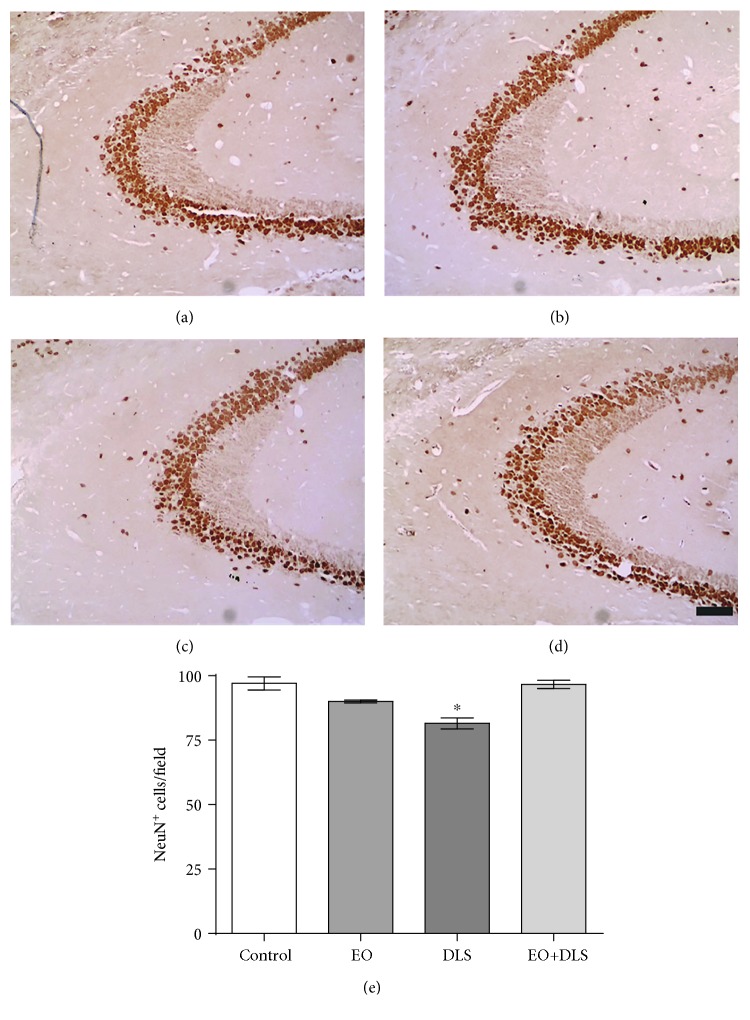
NeuN-positive cells in the CA3 region of the hippocampus of mice in a depressive-like state (DLS) and/or treated with clarified açaí (EO). Representative microphotographs of control (a), EO (b), DLS (c), and DLS+EO (d) groups and quantitation of NeuN-positive cells (e). Data are presented as mean and SD (*n* = 4‐5). ^∗^*P* < 0.05 vs. all groups. Scale bar: *μ*m.

**Figure 10:**
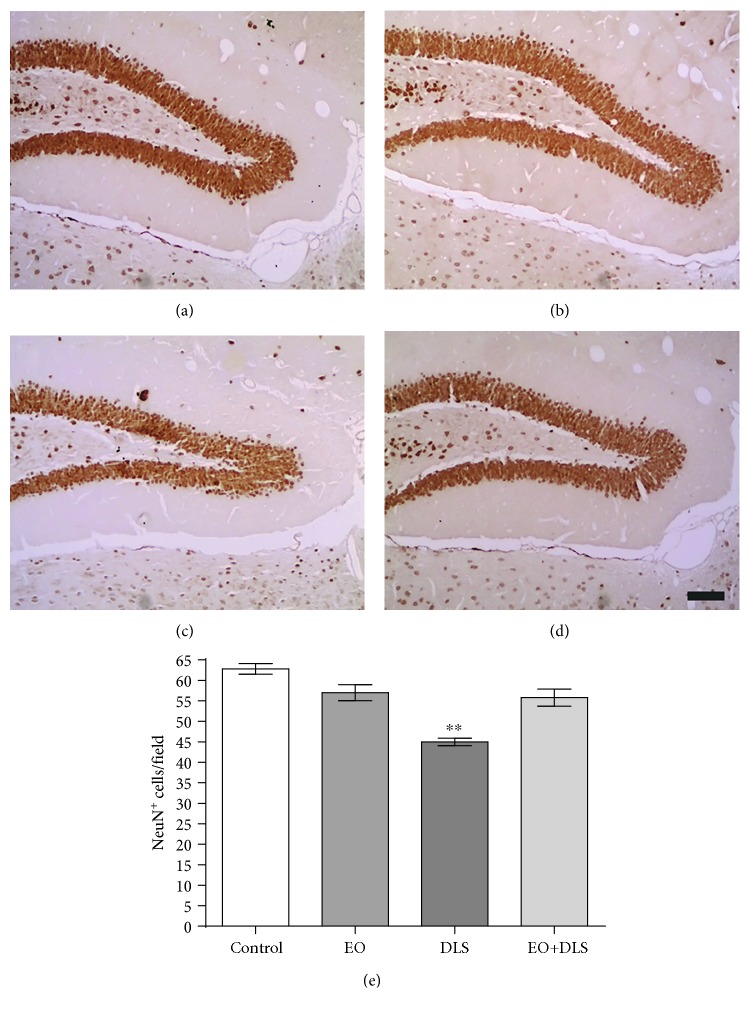
NeuN-positive cells in the dentate gyrus of mice in a depressive-like state (DLS) and/or treated with clarified açaí (EO). Representative microphotographs of control (a), EO (b), DLS (c), and DLS+EO (d) groups and quantitation of NeuN-positive cells (e). Data are presented as mean ± SD (*n* = 4‐5). ^∗∗^*P* < 0.01 vs. all groups. Scale bar: *μ*m.

Immunochemical studies in the hippocampus revealed a significant decrease in NeuN-positive cells in CA1 (Figure 8), CA3 (Figure 9), and dentate gyrus (Figure 10) of the animals presenting the depressive-like behavior. In all the three regions, EO protected the hippocampal cells, preventing the neuronal death associated with the DLS.

**Figure 11:**
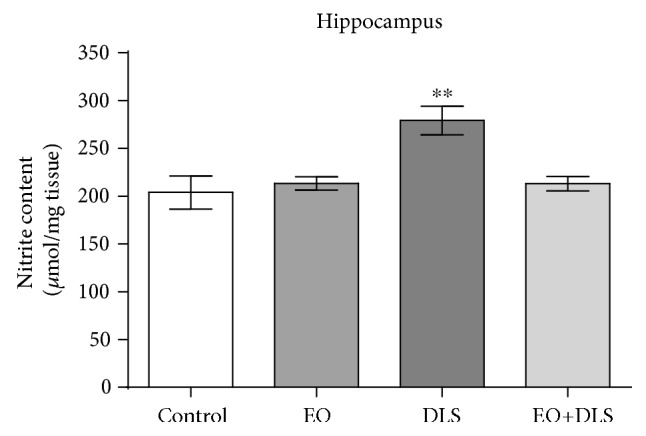
Nitrite levels in the hippocampus of mice in a depressive-like state (DLS) and/or treated with clarified açaí (EO). Data are presented as mean ± SD (*n* = 10). ^∗∗^*P* < 0.01 vs. all groups.

To confirm the important role of oxidative stress in the neuronal death detected in the hippocampus, we also measured nitrite levels as indirect markers of the production of one of the major free radicals, nitric oxide (Figure 11). Significantly high levels of nitrites were detected in animals with depressive-like behavior, pointing to an exacerbated nitric oxide production. This increase in products of this free radical was not observed in animals that received EO, demonstrating the potent antioxidant property of this fruit.

## 4. Discussion

This study is the first to demonstrate that EO juice for human consumption prevents the depressive-like behavior in an *in vivo* model. The potent antidepressant influence of EO was associated with antioxidant and antiaging effects in three major brain regions involved in mood regulation. Additionally, treatment with EO significantly protected hippocampal neurons and reduced the nitrite levels (an indirect marker of nitric oxide production).

Açaí juice is frequently consumed by Amazonian populations, and the clarified juice is available in the international market as a base for soft drinks [20]. In 2015, the State of Pará in Brazil alone consumed more than one million tons of açaí and exported more than 6 million tons of a mixture (açaí+banana+guaraná) to the United States and Japan [42]. The EO dose used in this work (equivalent to 750 mL for an adult of 70 kg, approximately) reproduces the daily intake of human populations in the northern region of Brazil (usually 300-1000 mL daily) [20]. This work tested for the first time commercial samples of EO (of known composition) in a model of depression, which guaranteed reproducibility of the preparation and relevance for human intake. Additionally, EO by gavage, not by free access to food, ensured that all animals received the same treatment.

In this study, we used a model of depressive-like behavior induced by the administration of a low dose of LPS (less than 20 times the dose used for endotoxemia models) [43]. The behavioral alterations in this model mimic some characteristics of clinical depression in humans [33, 44] and meet all the criteria (apparent, constructive and predictive validity) necessary for an animal model [45], being widely used for the screening of potential antidepressant drugs with good specificity and sensitivity [46]. Indeed, LPS exposure mimics a situation of increased plasma bacterial translocation. In line with this evidence, a recent study revealed that patients with depression present increased gut permeability and increased plasma levels of LPS when compared to unaffected people [19]. The presence of higher LPS plasma levels in patients with depression may trigger peripheral inflammatory and oxidative alterations by the activation of the Toll-like receptor 4. These cytokines may reach the brain, causing neuroinflammatory alterations and compromising neurotransmission mechanisms [47].

In this model, it is important to demonstrate the establishment of the depressive-like behavior with no symptom of sickness behavior (such as alterations in locomotor activity), because factors such as sex can influence the outcomes [48]. Our results demonstrated no alterations in the number of crossings and total distance covered in the open-field test, supporting the absence of sickness behavior in the LPS-treated animals (Figure 2). EO treatment alone did not change body weight (data not shown) or spontaneous locomotor activity (Figure 2), and it did not cause anhedonia/despair-like behaviors (Figures 3 and 4), confirming previous results regarding a lack of behavioral toxicity of the fruit [26].

Animals treated with LPS, however, presented much longer immobility in the forced swim test and consumed significantly less sucrose in the sucrose preference test (Figures 3 and 4). The latter tests are gold-standard methods for evaluating the depressive-like phenotype in rodents, especially to demonstrate the presence of despair-like and anhedonia behaviors [44, 49]. In our study, the increased immobility time and reduced sucrose consumption support the presence of a depressive-like phenotype in the animals. This study also applied reliable electromyographic measurements to confirm the DLS in the animals, an innovative method which may be more sensitive than behavioral testing alone (Figure 5).

Despite evident DLS in LPS-treated animals, the regular consumption of EO for 4 days was enough to eliminate all of these changes (Figures 3–5). Of interest, in the forced swim test, this protection was similar to that conferred by imipramine (Figure 3), pointing to a probable synergistic effect of EO and this drug (Figure 3, inset). Imipramine is a classical tricyclic antidepressant that blocks transport of monoamines, increasing serotonin and norepinephrine levels in the synaptic cleft. In addition to the modulation of the monoaminergic system, imipramine exerts antidepressant effects through its antioxidant activity [18]. In a similar way, the antidepressant effect of EO could be attributable to the inhibition of the monoaminergic system, in addition to potent antioxidant properties of the fruit. This hypothesis is supported by the modulatory effects that some açaí compounds (such as ellagic acid, ferulic acid, gallic acid, apigenin, rutin, and resveratrol) exert on the monoaminergic system [28, 29, 50–52]. Similar effects on the same targets could explain the apparent synergism observed in our work.

Although additional studies are necessary to confirm this possible synergism and their molecular mechanisms, our results are already promising for the treatment of depression because supplementation with EO may eventually allow for reduced doses of drugs with high toxicity, such as tricyclic antidepressants. Moreover, based on preclinical data, the protective effect of EO would be at least as potent as that of classical drugs used in depression, such as the SSRIs (fluoxetine and paroxetine), the serotonin-norepinephrine reuptake inhibitors (venlafaxine), or the tricyclic antidepressants (imipramine), as demonstrated by our results and those in the literature involving the same model [53, 54]. Although decreased serotoninergic and adrenergic actions were considered for many years to be the main explanation for depression and targets for drug development, other phenomena such as neuroinflammation, neurotrophism, or oxidative stress also play a major role and are targets for the development of new antidepressant therapies.

Among the most recent phenomena associated with MDD, an accelerated biological aging process has been related to clinical depression in epidemiological studies [3, 5, 6]. In these studies, aging was characterized by a significant decrease in telomere length and TERT expression in human peripheral and central nervous system cells [3–6]. Moreover, preclinical data demonstrated that inhibition of hippocampal TERT activity abolishes the behavioral effects of antidepressant drugs such as fluoxetine, supporting a close relationship between TERT activity and antidepressant effects [55].

In animal models of depressive-like behavior, reduced TERT expression has been reported in tissues such as the liver and hippocampus [55–57], but this study is the first to show a significant decrease in three major brain regions (the hippocampus, striatum, and prefrontal cortex) involved in depression (Figure 6). This pronounced decrease (between 25% to 50%) of the de novo synthesis of TERT may be partially responsible for the long-term deleterious consequences associated with depression, such as accelerated brain aging and increased susceptibility to age-related disorders. In our work, four doses of EO were enough to completely restore the expression of the enzyme (Figure 6), an effect similar to that described for treatments with classical antidepressants such as fluoxetine (10 mg/kg per day, for 28 days) [55]. This fact confirms the important role of TERT in the treatment of depression and the potency of the antidepressant effect of the fruit.

An interesting result was that EO alone could significantly increase TERT mRNA expression in these three brain areas, revealing a potent antiaging action of the fruit (Figure 6). The brain is especially susceptible to aging and age-related disorders, with serious effects on the quality of life of patients and their families. Additional studies will permit a better understanding of the antiaging effect of EO that we report here.

The presence of TERT is essential, especially in conditions of exacerbated oxidative stress, a known inducer of telomere shortening [58–60]. The oxidative profile of this model is largely known in the literature, with characteristics such as increased lipid peroxidation and decreased antioxidant defenses, especially involving glutathione ([18]; Tanaguti et al., 2019; Tanaguti et al., 2018). In fact, lipid peroxidation (evaluated using MDA levels) is frequently used in this model as the main hallmark of the consequences of oxidative stress on macromolecules ([18]; Domingues et al., 2018; Tanaguti et al., 2019; Tanaguti et al., 2018). In our work, animals in a DLS had significantly high levels of lipid peroxidation in the three studied brain areas (the hippocampus, striatum, and prefrontal cortex) (Figure 7), confirming earlier findings. Lipid peroxidation is frequently increased in both patients with depression [61] and in animals in a DLS ([33]; and this study).

The treatment with EO completely reversed this scenario, maintaining the levels of lipid peroxidation as low as those detected in the control group (Figure 7). This result is not surprising considering that an EO dilution of 1 : 100 has stronger scavenger properties than 800 *μ*M Trolox (a soluble analogue of vitamin E) [26]. This potent antioxidant activity of açaí is mainly exerted by anthocyanins, proanthocyanidins, flavonoids, and liganins [62], many of which have shown antidepressant properties singly in animal models [63–65]. Recent evidences demonstrated that phenolic compounds, such as anthocyanins, anthocyanidins, and orientin, improves depressive-like behavior [66, 67].

Considering the high potency of the antidepressant effect of the EO treatment observed in this work and the multiple targets that can be influenced by EO, such as GABAergic receptors and transporters [27], free radicals [26], or TERT expression (this study), it seems unlikely that the protective effect we have detected is related to only one compound. Probably, an association of several bioactive components contributes to this effect.

It is noteworthy that the oxidative stress especially affected the hippocampus, which presented about twice the levels of lipid peroxidation when compared to the other areas (Figure 7). This fact, in addition to the decreased TERT mRNA expression (Figure 6), suggests a specifically deleterious scenario for this area. TERT exerts a protective effect in the hippocampus, and hippocampal neurons lacking TERT have increased susceptibility to oxidative stress [68]. To study the possible hippocampal neuronal death in our model and the potential neuroprotection provided by EO, we carried out immunohistochemical analysis in three areas of the hippocampus (CA1, CA3, and dentate gyrus). In addition, the increased oxidative stress was confirmed by quantifying levels of nitrites, an indirect marker of nitric oxide production.

Our results showed that animals in a DLS had significantly decreased NeuN-positive cells in all three regions (Figures 8–10) and increased hippocampal levels of nitrites (Figure 11). Our data are in agreement with previous studies suggesting a prominent role of nitric oxide and the nitrergic pathway in the pathophysiology of depression and in the modulation of the behavioral and neurochemical changes observed in this model [33, 69].

The evident neuronal death detected in the hippocampus confirms the deleterious consequences of simultaneously increasing oxidative stress and decreasing TERT expression. The neuronal loss of the hippocampal regions (as high as 30.2%) would be in agreement with the reduced hippocampal volume observed in patients with depression [70, 71], apparently specific to the *cornu ammonis* and dentate gyrus areas [70], and responsible for some well-documented cognitive deficits (especially related to learning and memory) that accompany major depression [70, 71].

The potent neuronal protection observed with the EO treatment that completely abolished the hippocampal neuronal loss (Figures 8–10) suggests a general, if not universal, effect of EO for the brain. The treatment used in this work has already been suggested to protect neurons against other severe conditions, such as generalized seizures [26].

In summary, our results demonstrated an antidepressant effect of EO at different levels of analysis (behavioral, cellular, biochemical, and molecular). This potent effect (apparently, as potent as imipramine) was observed with only four doses of EO, similar in content to typical human consumption. Moreover, EO appeared to improve the effects of antidepressant drugs, such as imipramine, on depressive conditions. Our results, in addition to the absence of toxic effects for humans who consume similar amounts of the clarified juice [72], support the use of this fruit as an important protection for the brain against the development of depressive-like disorders. Moreover, for the first time, we describe an antiaging effect of EO that suggests neuroprotection against long-term age-related consequences.

## Data Availability

The data used to support the findings of this study are available from the corresponding author upon request.

## Abbreviations

EO: Clarified juice of *Euterpe oleracea*
DM: Dry matter
LPS: Lipopolysaccharide
TBARS: Thiobarbituric acid-reactive substance
MDA: Malondialdehyde.
